# Alterations in Resting-State Activity Relate to Performance in a Verbal Recognition Task

**DOI:** 10.1371/journal.pone.0065608

**Published:** 2013-06-13

**Authors:** Rocío A. López Zunini, Jean-Philippe Thivierge, Shanna Kousaie, Christine Sheppard, Vanessa Taler

**Affiliations:** 1 School of Psychology, University of Ottawa, Ottawa, Ontario, Canada; 2 Bruyère Research Institute, Ottawa, Ontario, Canada; University Medical Center Groningen UMCG, The Netherlands

## Abstract

In the brain, resting-state activity refers to non-random patterns of intrinsic activity occurring when participants are not actively engaged in a task. We monitored resting-state activity using electroencephalogram (EEG) both before and after a verbal recognition task. We show a strong positive correlation between accuracy in verbal recognition and pre-task resting-state alpha power at posterior sites. We further characterized this effect by examining resting-state post-task activity. We found marked alterations in resting-state alpha power when comparing pre- and post-task periods, with more pronounced alterations in participants that attained higher task accuracy. These findings support a dynamical view of cognitive processes where patterns of ongoing brain activity can facilitate –or interfere– with optimal task performance.

## Introduction

Resting-state brain activity is characterized by complex and highly non-random patterns of intrinsic activity generated while the brain is not actively involved in a task [1]. Electroencephalography (EEG) oscillatory patterns of resting-state activity are informative of the functional state of brain networks as well as their contribution to cognitive and behavioural performance [2], [3]. Despite much attention in recent work, a clear characterization of the links between resting-state before and after cognitive performance is lacking.

Task-induced oscillations obtained with EEG show that frequency-specific activity is associated with cognitive processes, including attention and memory [4]. Both theta (4–7 Hz) and alpha (8–12 Hz) power, for instance, have been associated with working memory processes [5], [6], [7], [8], [9] as well as selective attention [10], [11]. In addition, higher alpha power measured at baseline predicts subsequent learning rate during a game designed to study training strategies [12]. In a related study, the magnitude of change in alpha power from pre-task resting activity to task-related activity predicted episodic memory performance [13].

Findings relating task-induced oscillations to memory and attention have been complemented by studies examining patterns of resting-state activity that precede task involvement. Resting-state theta power, for instance, predicts subsequent verbal recall and attentional performance [14]. Resting-state alpha-band power, in comparison, predicts response accuracy in working memory tasks [15], [16] and memory performance during a free recall task [17]. Finally, higher alpha synchronization prior to stimulus presentation is predictive of the amplitude of event related potentials (N100) and is associated with faster reaction times [18]. These results suggest that resting-state activity preceding a task can be a reliable indicator of subsequent cognitive functions that are relevant to information processing in the brain.

In addition, resting-state brain dynamics form an ongoing process that is highly plastic and influenced by cognitive demands and learning [19], [20]. Therefore, in the present study, we investigated the resting-state activity before and after a task of verbal recognition. We monitored EEG activity in delta, theta, alpha, beta and gamma bands before and after the task. We begin by examining behavioural performance (response times and accuracy) in relation to pre-task resting-state oscillations. Next, we compare the power of pre- and post-task resting-state activity, addressing whether behavioural performance reshapes the statistics of resting-state oscillations.

## Methods

### Ethics Statement

All participants gave written informed consent and were paid for their participation. The study protocol was approved by the Bruyère Research Institute Research Ethics Board and in accordance with the Code of Ethics of the World Medical Association (Declaration of Helsinki).

### Participants

Twenty-eight participants were recruited for the study. All participants were young adults randomly recruited from the University of Ottawa. Prior to testing, participants completed a health history questionnaire. All participants were native English speakers, with good self-reported health, normal or corrected-to-normal vision, and no neurological or psychiatric history. No participant was taking any medications known to affect cognitive function. Participants were randomly assigned to either an experimental condition (“Task Group”) or a control condition (“No-task Group”; *n = *14 participants per group). Task and no-task groups did not differ in age or education (average age = 22.34+/−1.78, *p*>.71; average education = 15+/−1.61, *p*>.14).

### Task Group

For participants in the Task Group, resting EEG activity was recorded for 10 minutes immediately prior to performing a verbal recognition task (see below), during the verbal recognition task (approximately 5 min in duration), and for 10 minutes immediately afterwards. During recordings of resting-state EEG, participants were instructed to remain relaxed and look at a white fixation point on black background on a computer screen. They were instructed to minimize blinking and a research assistant monitored for excessive blinking or horizontal eye movements by visual inspection of EEG during recording. Participants were informed that, in the event of excessive eye movements, the research assistant would remind them to fixate their gaze on the fixation cross. Participants were compliant and did not produce excessive eye movements during recording; reminders were therefore not employed.

#### Verbal recognition task

**Figure 1:**
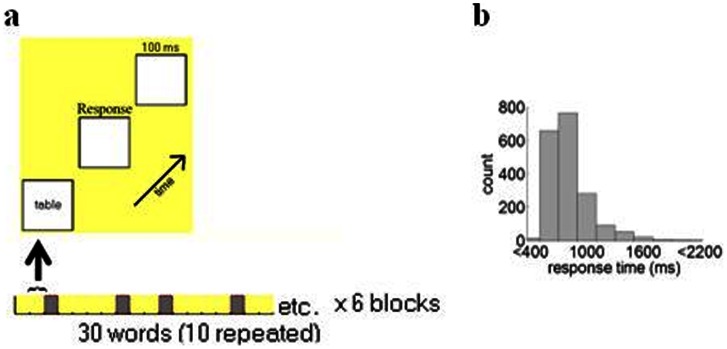
Verbal recognition task and reaction time distribution. **a.** Top: Illustration of a single word stimulus. A word (e.g.,“table”) appeared on the screen and remained there until the participant provided a response. Immediately following the participant’s response, the screen remained blank for 100 milliseconds before the next word was presented. Bottom: design of a single block in the verbal recognition task. Each block comprised novel (yellow) and repeated words (gray). The complete task featured 6 blocks of 30 words each (10 novel and 10 repeated words). **b.** Distribution of response times across all participants and trials. Arrow: overall mean.

Participants in the Task Group performed a speeded verbal recognition task in which they viewed words one at a time on a computer screen and were required to decide whether or not the word had appeared previously in the list. The task included a total of 180 words (e.g. money, fruit, window) divided into 6 blocks (Fig. 1a, bottom). Each block comprised a list of 30 words, where 10 words were randomly repeated within the list. Thus, in each block, there were 20 unique words, and 10 of these words were repeated a second time within the block. The mean repetition lag for words (i.e. number of words between the first and second presentation of the repeated words) was 9 (range: 1 to 20).

Stimuli in each list were controlled for length and frequency using norms from the Celex database [21]. Participants indicated whether they had seen each word previously in the list or not by pressing a button on the computer keyboard (the “l” key for “yes” responses and the “a” key for “no” responses). Participants kept their hands on the keyboard and used their left hand to press for “a” and their right hand to press for “l”. Each word appeared at the center of the screen in black bold 18-point Courier New font on a white background with 100 ms inter-stimulus interval (Fig. 1a, top). Words remained on the screen until the participant provided a response. After this response, a 100 ms inter-stimulus interval preceded the onset of the next stimulus. The task took approximately 5 minutes to complete and was run using E-prime (Psychological Software Tools, Inc., Sharpsburg, Pennsylvania).

### No-task Group

Testing was identical for participants in the No-task Group (control condition), except that rather than completing the word recognition task, they were instructed to relax and remain seated for a 5-minute interval corresponding to the duration of the task.

### Data Acquisition and Pre-processing

EEG signals were recorded continuously from six midline sites and 23 lateral sites according to the international 10–20 system of electrode placement using a nylon EEG cap containing tin electrodes (Electro-Cap International, Inc., Eaton, OH, USA). A cephalic (forehead) location was used as a ground and all active sites were referenced on-line to linked ears using Scan 4.3 computer software (Neuroscan, El Paso, TX, USA). We recorded the horizontal electro-oculogram (EOG) as bipolar channels from electrodes placed at the outer canthi of both eyes and the vertical EOG from electrodes placed above and below the left eye. EEG signals were amplified using Neuroscan NuAmps (Neuroscan, El Paso, TX, USA) and acquired at a sampling rate of 500 Hz in a DC to 100 Hz bandwidth with electrical impedances <8 kΩ. Vertical EOG artefacts were corrected off-line using a spatial filter (Neuroscan, EDIT4.3) and trials with horizontal EOG artefact exceeding peak amplitudes of 50 µV were excluded. EEG trials containing deflections exceeding 100 µV were also excluded. Finally, EEG recordings were visually inspected and trails containing movement artifacts were manually removed. After removal of eye blinks and artifacts during resting state, an average 8 minutes of EEG recording remained for each participant.

### Data Analyses

#### Behavioural data analyses

Mean accuracy and reaction time were calculated for each participant. Behavioural trials where performance accuracy and reaction time were identified as outliers were removed on a participant-by-participant basis. This was done by calculating the mean accuracy and reaction time for each participant. Outliers, defined as trials where the response time and/or accuracy was greater than 2.5 standard deviations from the participant’s mean, were removed (an average of 1.5 trials per participant were removed, over a total of 180 trials).

#### EEG data analyses

All EEG data were analyzed using custom software written in the Matlab language (Mathworks Inc., Natick, Massachusetts). Since an average of 8 minutes of resting state activity remained after artifact removal, each participant was left with different durations of resting activity. To insure that the same amount of resting state data were considered for each participant, we restricted our FFT analysis to the last 5 minutes of data for each participant and each recording period (pre- and post-task). After removal of movement artifact, segments of different lengths (over the last 5 minutes of data) were used to compute EEG power (µv^2^).

For each electrode, the mean power spectrum across participants was normalized between 0–1 [22]. Power was then averaged across delta (1–3.5 Hz), theta (4–7.5 Hz), alpha (8–12.5 Hz), beta (13–29.5 Hz) and gamma (30–100 Hz) bands (Fig. 1b).

In order to estimate changes in EEG power between pre- and post-task recordings, we computed, for the Task and No-task groups separately, the best-fitting linear regression relating the average band-specific power (mean power over all electrodes) for each participant for pre- vs. post-task resting periods. We did this in the following way: first, we computed the mean alpha power across electrodes for each participant within a given group. Second, we fitted a regression line relating pre- and post-task power across all participants of the group. Third, we computed the residual error of the linear regression for each participant. This provided us with an estimation of change in alpha power (Δalpha) between pre- and post-task periods that removed linear drifts known to occur in EEG activity over time, an analysis that is analogous to linear detrending [23]. Linear detrending, however, is typically performed on a per-subject basis, whereas the Δalpha analysis is performed at the group level.

A paired t-test was used to compare Δalpha for the Task vs. No-task groups (statistical criterion of α = .01). In a follow-up analysis, we calculated Δalpha for each electrode individually in order to show the topographic distribution of alterations in alpha power. Finally, we performed correlations between Δalpha for each electrode and performance accuracy using the surrogate data approach (see surrogate subsection for details).

Surrogate data approach**.** Electrode-by-electrode analyses were conducted to relate resting-state EEG power to measures of task performance. We implemented an approach based on surrogate data in order to identify electrodes whose band-delimited power correlated with task performance [24]. The goal of this approach is to compare Pearson correlations between EEG power and task performance with correlations obtained using random EEG data that share basic properties of the original data (in terms of mean, variance, and Fourier spectrum). Higher correlations in the original data compared to those of random data are considered statistically above-chance. This approach allows for the estimation of correlation values that would occur by chance merely due to the high number of comparisons being carried [25]. Values above the chance correlation threshold that is obtained by our surrogate approach are considered statistically significant.

Formally, we can represent a given measure of EEG oscillations (e.g., alpha power) as a matrix of size *M*x*N* where *M* is the number of electrodes and *N* is the number of participants, while performance can be represented as a row vector **y** = 1,…,*N* containing a single value (e.g., average accuracy) per participant:
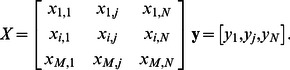
(1)


A series of correlations are performed between **y** and rows of *X* in order to compare alpha power and task accuracy for each individual electrode:
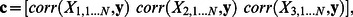
(2)where 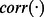 denotes Pearson correlation.

We wish to establish a statistical criterion for each element of **c** that will identify above-chance correlations between power and task performance. Here, we compute this statistical criterion independently for each electrode using a method of surrogate data. The goal of this method is to generate artificial EEG data (herein referred to as surrogate data) that share properties of the original data (including mean, variance, and Fourier spectrum) but is otherwise random. The correlation between artificial EEG data and participants’ task performance is informative of the magnitude of random effects, and can be employed to derive a statistical criterion as described below.

Surrogate EEG data were generated using the amplitude adjusted Fourier transform (AAFT) algorithm [26]. This algorithm examines the original EEG data and outputs random EEG data that closely approximates the mean, variance, and Fourier spectrum of the original data. The AAFT algorithm works as follows. Let 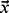 be an EEG timeseries for a single electrode with elements *t* = 0,…,*N*-1. First, a Gaussian timeseries 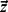 is produced, where each element is independently drawn from a Gaussian distribution with µ = 0 and σ = 1. Then, the vector 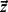 is reordered so that the ranks of 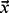 and 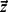 agree. In other words, if 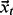 is the *n* ^th^ smallest value in 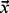, then 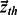 will be the *n* ^th^ smallest value in 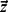. As a result, both time series will follow each other and their amplitude will follow a Gaussian distribution.

Given a timeseries 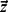, data surrogates 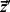 are generated as follows. First, an unwindowed Fourier transform is computed for positive and negative frequencies *f* = 0,1/*N*,2/*N*,…,1/2. Second, the phase of the timeseries is randomized by multiplying the complex amplitude at each frequency by *e^i^* ^φ^, where the phase φ is chosen independently for each frequency from the interval [0,2π]. Third, the phases are symmetrized so that φ(*f*) = –φ(–*f*). Finally, surrogate data 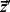 is generated as the inverse Fourier transform. Surrogate data generated in this way has gaussian amplitude and matches the Fourier spectrum of the original data. Assignment of the surrogate timeseries was randomized across participants (within-group), following the null hypothesis that task performance across participants was unrelated to their resting EEG activity.

Surrogate timeseries were obtained for each electrode of each participant. Alpha power of the surrogate timeseries were computed, yielding a matrix 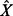 that is the random homologue of 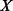
in Eq.1. We then applied Eq.2 after substituting 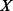
for 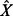, yielding a vector of correlations 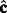
between surrogate data and mean task accuracy.

The above process of generating surrogate data and computing its correlation to task performance is repeated a total of 100 times. Correlations between surrogate data and mean task accuracy were stored in a matrix 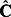
of size *M*x*Q* where *M* is the number of electrodes and *Q* is the number of independently-generated surrogate datasets.

Finally, we computed a significance threshold for each electrode *j* such that its value exceeded that of at least 99% of columns in 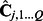. If this threshold was lower than the correlation between task accuracy and alpha power in the original data, we considered that the latter correlation was above chance.

**Figure 2:**
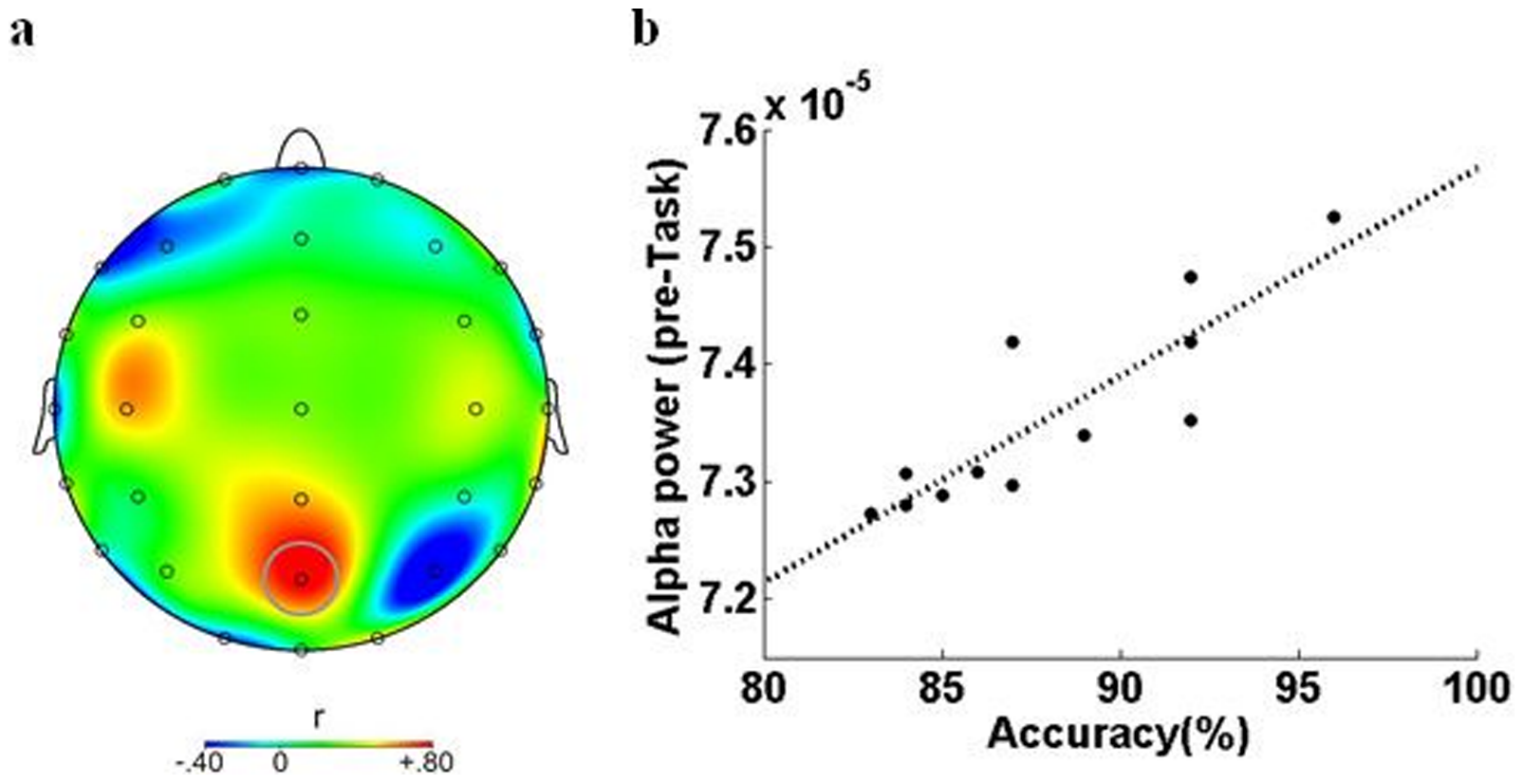
Relationship between pre-task resting-state alpha activity and task accuracy. **a.** Topographic map showing electrode-by-electrode correlation values between alpha power (µv^2^) and mean performance accuracy (% correct responses) averaged over all participants (spline interpolation between sites). Gray circle: electrode Pz showing highest correlation value. **b**. Positive correlation between pre-task alpha power (electrode Pz) and percentage accuracy (r = .86, p<.0001). Black dots show individual participants. Dashed line is the best-fitting (least squares) linear regression.

**Figure 3:**
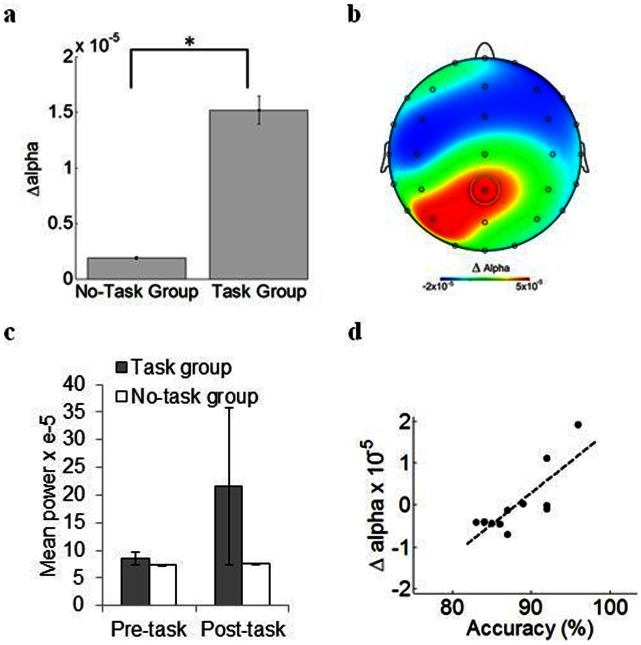
Alterations in resting-state alpha power following the verbal recognition task. **a.** Average change in alpha power (Δalpha) between pre- and post-task resting-state periods (see Methods). Vertical bars = SEM. *p<.001. **b.** Topographic distribution of Δalpha values, showing that posterior parietal electrodes had strongest alterations in alpha power between pre- and post-task resting-state periods. Colorbar indicates mean Δalpha power values (in µv^2^) averaged over participants. Gray circle: electrode CPz (highest Δalpha value). **c.** Mean alpha power pre- and post-task for the Task and No-Task groups at electrode CPz. Vertical bars = SEM. For the No-task group, “post-task” activity refers to resting-state activity recorded after a 5 min waiting period corresponding to the duration of the task. **d.** Positive correlation between mean accuracy (% correct responses) and change in alpha power (Δalpha) for site CPz. Black dots show individual participants. Dashed line is the best-fitting (least squares) linear regression.

A tutorial and code for surrogate data approach can be found at https://sites.google.com/site/jpthivierge. Raw EEG data in Matlab format is available from the corresponding author. Topographic maps shown in figures 2–3 (described below) were generated with BrainVision Analyzer 2.0 (Brain Products, Morrisville, USA) using spline interpolation. These maps show the correlation values between performance measures and EEG power for each electrode.

## Results

### Resting-state Activity Predicts Task Performance

EEG activity was collected before and after the verbal recognition task. Measures of behavioural performance included responses times and accuracy in the identification of repeated words. Response times were similar to previously reported findings for similar tasks, with a mean reaction time of 823.30 ms (s.d. 109.25 ms) [24] (Fig. 1b). The overall mean accuracy was 88% (s.d. 4%). The reaction time and accuracy of responses to novel and repeated words are shown in Table 1. Results revealed that accuracy was higher for novel than repeated words (one-way repeated-measures ANOVA, F(1,11) = 1.30, p<.01). In addition, incorrect answers yielded slower reaction times than correct answers (F(1,12) = 25.72, p<.001).

**Table 1:** Mean reaction time and accuracy during the verbal recognition task.

Stimulus Type	Mean Reaction Time in ms (s.d.)	Mean % Accuracy (s.d.)	
*Correct Answers*			
Novel Words	811.97(128.97)	93(4)	
Repeated Words	820.84(110.52)	78(13)	
*Incorrect Answers*			
Novel Words	1407.73(443.28)		––
Repeated Words	1110.38(450.33)		––

The first question we sought to address was whether resting-state EEG activity immediately preceding the verbal recognition task was predictive of performance accuracy and reaction time. In a first series of analyses, we compared the band-delimited power of resting-state EEG obtained for each participant prior to the task with their mean reaction time and accuracy.

For each individual electrode, we computed the Pearson correlation between mean resting-state EEG power and mean performance accuracy (% correctly identified stimuli as either repeated or novel) across participants. Correlation values across electrodes ranged from r = −.61 to r = .86. The statistical significance of correlation values was assessed with the surrogate data approach based on 100 independently-generated surrogate data sets, each set containing 12 “surrogate” participants (see Methods). We uncovered a statistically reliable predictor of accuracy, namely alpha power at posterior midline (parietal) electrode Pz (*r* = .86; 99% correlation threshold based on surrogate data: *r* = ±.74). We also found a strong link between both beta and gamma power at Pz and task accuracy (See Table 2).

**Table 2:** Correlations at electrode Pz for each frequency band.

Frequency Bands	Correlation (r)	Surrogate threshold	p-value
Delta (1–3.5 Hz)	.50	±.72	.1
Theta (4–7.5 Hz)	.62	±.81	.03
Alpha (8–12.5 Hz)	.86	±.74	.0001
Beta (13–29.5 Hz)	.74	±.73	.005
Gamma (30–100 Hz)	.75	±.68	.004

To further control for differences in the number of trials separating two repeats of the same word, we performed the following analysis. First, we divided trials involving repeated word into repeated words with short lags (i.e., between 1 and 9 trials between two repeats of the same word) and repeated words with long lags (i.e., between 10 and 20 trials). Next, we computed separate Pearson correlations to compare EEG activity with either (1) mean accuracy on trials with short lags between repeated words; or (2) mean accuracy on trials with long lags. We focused these analyses on resting state alpha power at electrode Pz.

Results showed that, on trials with a short lag between repeated words, accuracy was positively correlated with pre-task resting state activity at Pz (r = .72, p<.009). Similarly, on trials with a long lag between repeated words, accuracy was positively correlated with pre-task resting state activity at Pz (r = .73, p<.007). These results support our above finding that resting state alpha power at Pz correlates with performance accuracy and argue that resting state predicts performance accuracy for trials with both short and long delays between repeated words.

These results support a link between posterior parietal resting-state activity and performance accuracy, as participants with higher resting-state alpha power attained higher levels of accuracy.

The relation between pre-task resting-state EEG and reaction times was evaluated using a similar analysis as above. No statistically significant correlation was found between resting-state EEG power and reaction times in any of the frequency bands considered (delta, theta, alpha, beta, or gamma), suggesting that pre-task resting-state EEG is not a strong predictor of response times. This finding is robust to changes in the statistical criterion (95%, 98%, or 99% threshold based on surrogate method), as all correlation values were markedly low (correlation values ranging from *r* = −.06 to *r* = .24).

Taken together, the above results suggest a link between resting-state alpha power at posterior sites and accuracy in the verbal recognition task. Next, we consider whether resting-state activity undergoes any systematic changes following the verbal recognition task, and whether these changes are related to performance on the task.

### Alterations in Subsequent Resting-State Activity Related to Task Performance

A 2×2 ANOVA with Resting period (two levels: pre-task and post-task) and Group (two levels: task and no-task) as factors was first performed. Results of this analysis revealed no significant interaction between Resting period and Group (F(1, 24) = 2.34, p>.14). There was also no significant main effect of Resting period (F(1, 24) = 3.11, p>.09) or Group (F(1, 24) = .14, p>.70). Upon further inspection, we found that, following the task, alpha power decreased for some participants, and increased for others. ANOVA may not detect a consistent trend if alterations in EEG did not occur in the same direction across participants. Given this variability, we performed a second series of analyses as follows. To examine alterations in resting-state activity after completion of the task, we computed a measure of Δalpha, which estimates changes in alpha power between pre- and post-task resting-state periods (see Methods). Values of Δalpha were compared between participants who performed the verbal recognition task (Task group) and a control group that did not perform the task (No-task group).

Participants in the Task group exhibited markedly higher values of Δalpha than participants in the No-task group (paired sample *t*-test, *t*(11) = 3.28, *p*<.0007) (Fig. 3a). This effect was largest at central parietal electrode CPz (Fig. 3b). Mean alpha values for CPz in Fig. 3c.

The above analysis comparing Δalpha for the Task versus No-task groups was repeated for all frequency bands tested (delta, theta, beta, and gamma), using Bonferroni-corrected paired sample t-tests (statistical criterion of α = .05/5 = .01 corrected for multiple bands). No significant differences between the Task and No-task groups were found for bands other than alpha (see Table 3). Task-related alterations in resting-state activity were thus specific to alpha power.

**Table 3:** Mean fluctuation values (Δ) values for the Task and No-Task Group in each frequency band.

Frequency Bands	Fluctuation (Δ values×e^−5 ^in µv2)		p-value^a^
	*Task Group*	*No-Task Group*	
Delta (1–3.5 Hz)	2.96 (2.81)	7.57(11.8)	.17
Theta (4–7.5 Hz)	.95 (.88)	.63(.58)	.28
Alpha (8–12.5 Hz)	1.32(1.18)	.18(.19)	.0007**
Beta (13–29.5 Hz)	.41(.42)	.16(.11)	.07
Gamma (30–100 Hz)	.16(.22)	.082(081)	.25

a Bonferroni corrected statistical criterion of **p = .05/5 = .01.

Are the above changes in subsequent resting-state alpha power related to task performance? To address this question, we began by computing the correlation between Δalpha at each electrode and performance accuracy (percentage of words correctly identified as either repeated or novel). We then evaluated the statistical significance of the resulting correlation values with the surrogate data approach (see Methods). Performance accuracy was robustly correlated with Δalpha at central parietal electrode CPz (*r* = .81, correlation threshold based on surrogate data: *r* = ±.72), suggesting that alterations in resting-state alpha power at CPz are related to performance accuracy (Fig. 3d). The correlation between Δalpha and accuracy seemed strongly driven by the two participants with highest values of Δalpha. A follow-up correlation excluding these participants, however, still yielded a robust correlation between alpha-band alterations and task accuracy (*r* = .67, *p*<.03). These results provide supporting evidence for task-related changes in the statistics of resting-state activity. More pronounced alterations in central parietal alpha power (as measured by Δalpha) were associated with higher performance accuracy.

In summary, participants’ resting-state alpha power was altered after performing the task. These alterations were markedly greater than in those participants who entered the control condition and hence did not perform the task. This result was specific to alpha power, and was not replicated for other frequency bands. Lastly, the degree of alteration in alpha power at electrode CPz was strongly associated with response accuracy, with more pronounced alterations in participants who correctly identified a larger number of words.

## Discussion

Our goal was to investigate the relationship between resting-state EEG and performance during a verbal recognition task in which participants identified repeated and novel words. Participants whose pre-task alpha-band resting-state activity was higher attained greater accuracy in verbal recognition and this effect was topographically delimited to a central-posterior (Pz) site.

We also investigated resting-state activity immediately following task execution and found marked alterations in alpha power that were positively linked to performance: participants with larger alterations in alpha power (localized to central parietal site CPz) responded with greater accuracy.

### Alterations in Alpha Activity are Related to Behavioural Performance

Our results go beyond previous work linking resting-state alpha activity with heightened performance [27]. We show that resting-state activity does not simply return to baseline levels after task execution; rather, involvement in the task alters alpha-band resting-state activity. Furthermore, higher task accuracy is associated with more pronounced changes in alpha-band activity. One possible explanation is that alterations in resting-state dynamics reflect state transitions (i.e., coordinated changes in activity) triggered by participants’ behavior during the task [28]. Independently of the underlying mechanism, the fluctuations in alpha power we observed between pre- and post-task periods clearly suggest that resting-state activity may be modulated by the demands of a cognitive task.

Recent research has indicated that structural plasticity may occur after several training sessions [19] or even hours of practice [29]. However, the changes in resting state activity reported here may be too rapid to reflect such plasticity. It is also unlikely that alterations in resting state activity can be accounted for in terms of spontaneous fluctuations in EEG signals [30] because pronounced alterations in alpha power were not observed in the control (No-task) group.

Nevertheless, it is important to acknowledge that alterations observed in the task group could be related to factors such as fatigue, the passage of time, and learning the task. It may be possible to elucidate the causes of change in alpha activity with the addition of control groups that perform a low/medium demand task such as merely observing words on the screen or reducing the repetition lag between words.

### Resting-state and Task-related Activity

Research shows that the larger the change in alpha power from resting to performance during a picture memory task, the better the performance during recall [13]. Here, we add to such findings by showing that alpha-band resting-state activity before a verbal recognition task can predict accuracy during the task. In addition, we compared alpha resting-state activity pre- and post-task and found that alterations in resting-state activity also related to performance accuracy. Studies show that higher alpha power during task performance is related to event-related potential (ERP) components that predict sustained attention [31], One promising avenue would be to examine the link between ERPs and resting-state activity [27] in a way that would integrate ERP components related to cognitive operations with band-delimited EEG activity.

Alternative measures of ongoing EEG fluctuations could also be considered, including coherence both within and across sites [32] as well as cross-frequency coupling [33]. While our findings did not find a link between response times and resting-state activity, perhaps these alternative measures could help draw a more complete portrait of the complex links between ongoing brain fluctuations and online behavioural measures.

While the current study examined resting-state EEG with opened eyes, further work will elucidate whether a stronger link to accuracy can be obtained with eyes opened. Resting-state EEG differs between eyes closed and opened conditions [34], with a stronger relation reported between memory performance and alpha power with closed eyes compared to opened eyes [17]. It remains unknown whether these differences will extend to pre- versus post-task alterations in resting state activity as reported here.

It is unclear at present whether inter-participant differences in the statistics of resting-state activity remain stable over repeated testing sessions. Ongoing alpha activity is known to exhibit multistable states (consistent fluctuations in patterns of activity) that continuously alternate between low and high amplitudes [28]. The time course of these states may account for inter-individual differences before and after task execution. Under this account, participants in a state of high alpha power at rest would perform more accurately than participants in a state of low alpha power, as we found. Alternatively, differences in alpha power across participants may reflect attributes of brain activity that are stable over extended time periods, a question that remains to be addressed.

The ubiquity of resting-state activity across brain states and anatomical regions suggests that it plays a fundamental role in brain function and cognitive processes. The present study shows that resting-state activity predicts performance accuracy during a verbal recognition task; and that performance during the task reshapes the patterns of resting-state activity. The presence of a mutual relationship between cognition, resting-state activity and alpha power during the task raises fundamental questions about the emergent nature of mental processes – questions that are gradually being addressed by exploring the dynamical properties of ongoing brain networks.
